# Supplementary material for: Curated and harmonised transcriptomics datasets of interstitial lung diseases
Source: Data Brief. 2025 Oct 14;63:112139. doi: 10.1016/j.dib.2025.112139 (PMC12581653; doi:10.1016/j.dib.2025.112139)
Supplement: Supplementary file 1 [file mmc1.zip › Supplementary_material/DNA-microarray/GSE10667/GSE10667_eUTOPIA_Agilent_QC_Report_2024-02-15.tar.gz!/Users/OWNER/AppData/Local/Temp/RtmpspbZX7/2024-02-15/out box.pdf]

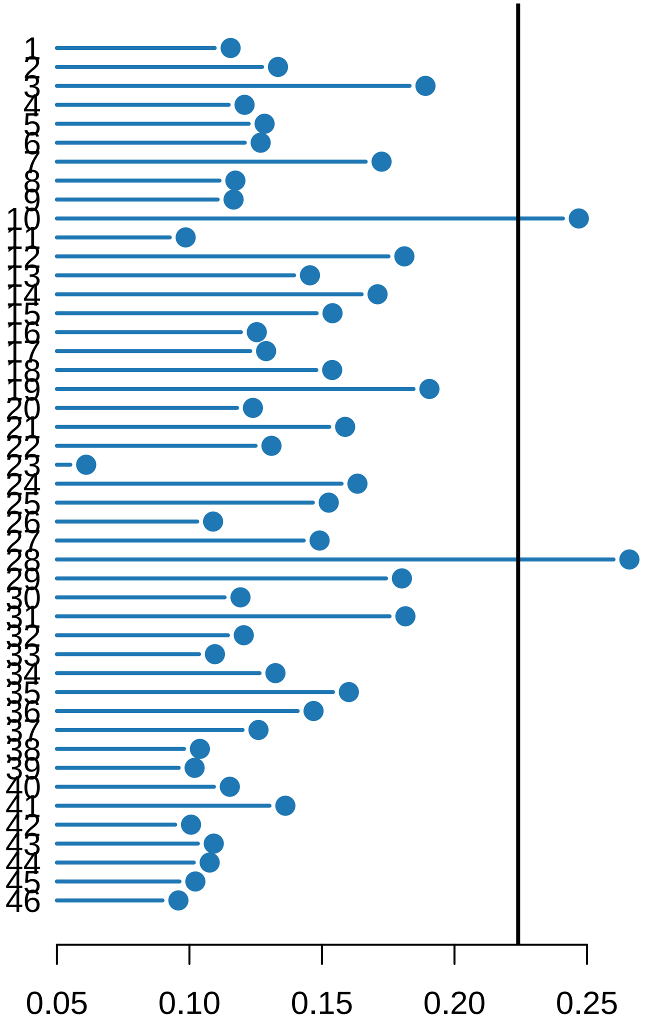
1
2
3
4
5
6
7
8
9
10
11
12
13
14
15
16
17
18
19
20
21
22
23
24
25
26
27
28
29
30
31
32
33
34
35
36
37
38
39
40
41
42
43
44
45
46
0.05
0.10
0.15
0.20
0.25
